# Supplementary material for: Novel Screen-Printed Sensor with Chemically Deposited Boron-Doped Diamond Electrode: Preparation, Characterization, and Application
Source: Biosensors (Basel). 2022 Apr 13;12(4):241. doi: 10.3390/bios12040241 (PMC9027657; doi:10.3390/bios12040241)
Supplement: Supplementary file 1 [file biosensors-12-00241-s001.zip › biosensors-1667856-supplementary.pdf]

biosensors

Supplementary Materials

Novel Screen-Printed Sensor with Chemically Deposited Boron-Doped Diamond Electrode: Preparation, Characterization, and Application

Oleksandr Matvieiev [1], Renáta Šelešovská [1,*], Marian Vojs [2], Marián Marton [2], Pavol Michniak [2], Vojtěch Hrdlička [3], Michal Hatala [4], Lenka Janíková [1], Jaromíra Chýlková [1], Jana Skopalová [5], Petr Cankař [6] and Tomáš Navrátil [3,*]

[1] Institute of Environmental and Chemical Engineering, Faculty of Chemical Technology, University of Pardubice, Studentská 573, 532 10 Pardubice, Czech Republic; oleksandr.matvieiev@student.upce.cz (O.M.); lenka.janikova@upce.cz (L.J.); jaromira.chylkova@upce.cz (J.C.)
[2] Institute of Electronics and Photonics, Faculty of Electrical Engineering and Information Technology, Slovak University of Technology in Bratislava, Ilkovičova 3, 812 19 Bratislava, Slovakia; marian.vojs@stuba.sk (M.V.); marian.marton@stuba.sk (M.M.); pavol.michniak@stuba.sk (P.M.)
[3] J. Heyrovsky Institute of Physical Chemistry of the Academy of Sciences of the Czech Republic, Dolejškova 3, 182 23 Prague, Czech Republic; vojtech.hrdlicka@jh-inst.cas.cz
[4] Department of Graphic Arts Technology and Applied Photochemistry, Faculty of Chemical and Food Technology, Slovak University of Technology in Bratislava, Radlinského 9, 812 37 Bratislava, Slovakia; michal.hatala@stuba.sk
[5] Department of Analytical Chemistry, Faculty of Science, Palacký University in Olomouc, 17. listopadu 1192/12, 779 00 Olomouc, Czech Republic; jana.skopalova@upol.cz
[6] Department of Organic Chemistry, Faculty of Science, Palacký University in Olomouc, 17. listopadu 1192/12, 779 00 Olomouc, Czech Republic; petr.cankar@upol.cz
* Correspondence: renata.selesovska@upce.cz (R.Š.); tomas.navratil@jh-inst.cas.cz (T.N.); Tel.: +420-466-038-053 (R.Š.); +420-266-053-886 (T.N.)

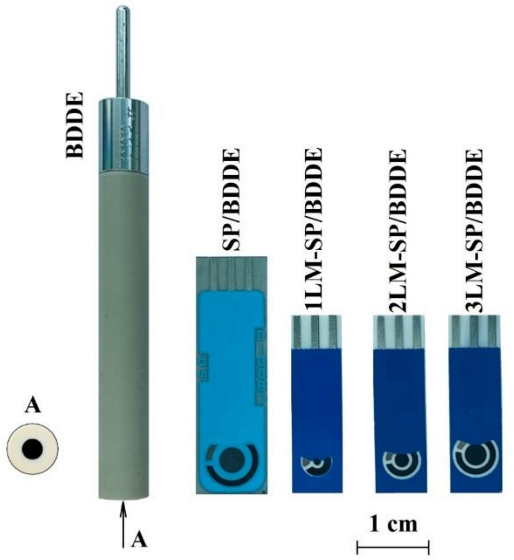

Figure S1. Applied sensors.

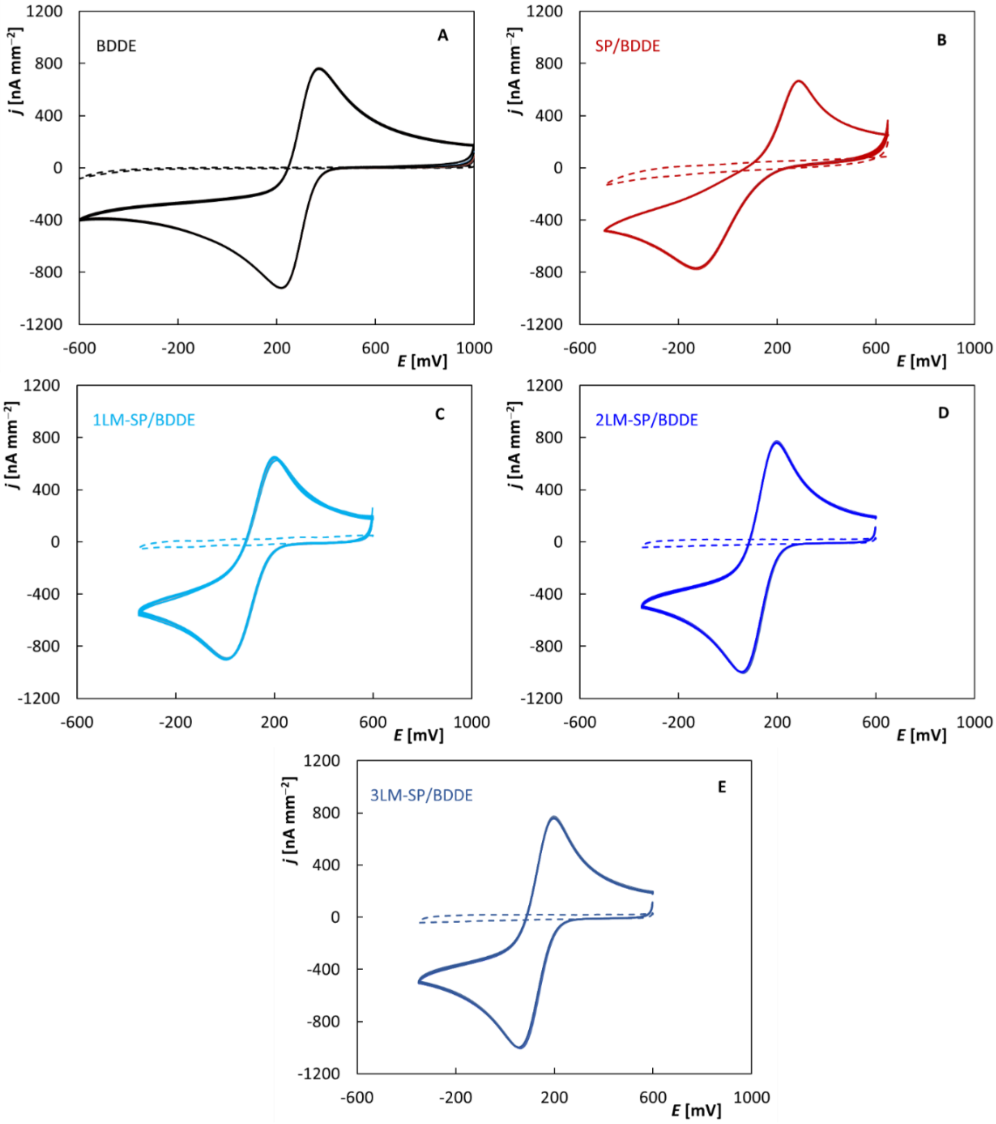

Figure S2. Cyclic voltammograms of $[Fe(CN)_6]^{4-/3-}$ recorded on all tested sensors (electrolyte – 0.1 mol L^{-1} KCl, v = 100 mV s^{-1}, $c([Fe(CN)_6]^{4-/3-})$ = 6.25×10^{-4} mol L^{-1}).

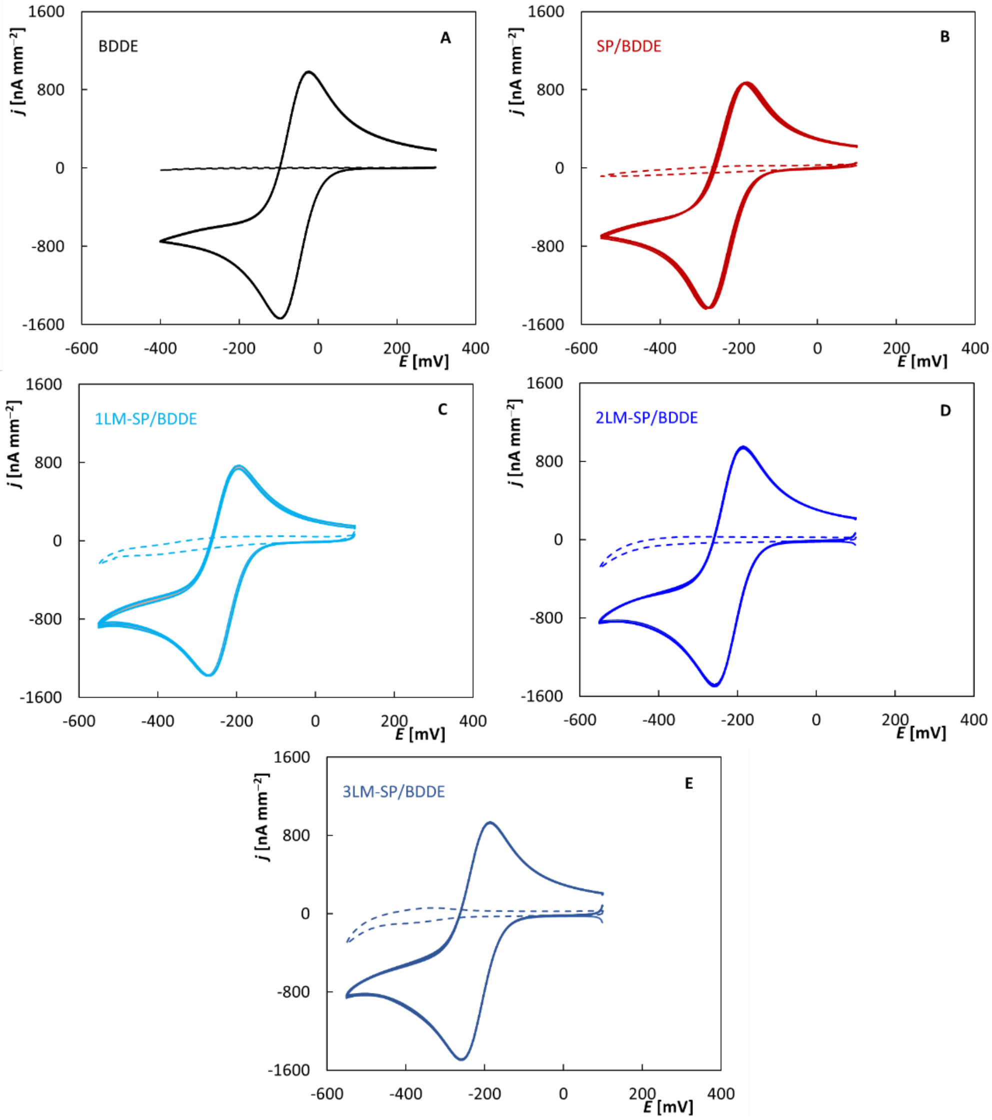

Figure S3. Cyclic voltammograms of $[Ru(NH_3)_6]^{2+/3+}$ recorded on all tested sensors (electrolyte – 0.1 mol L^{-1} KCl, v = 100 mV s^{-1}, $c([Ru(NH_3)_6]^{2+/3+})$ = 6.25×10^{-4} mol L^{-1}).

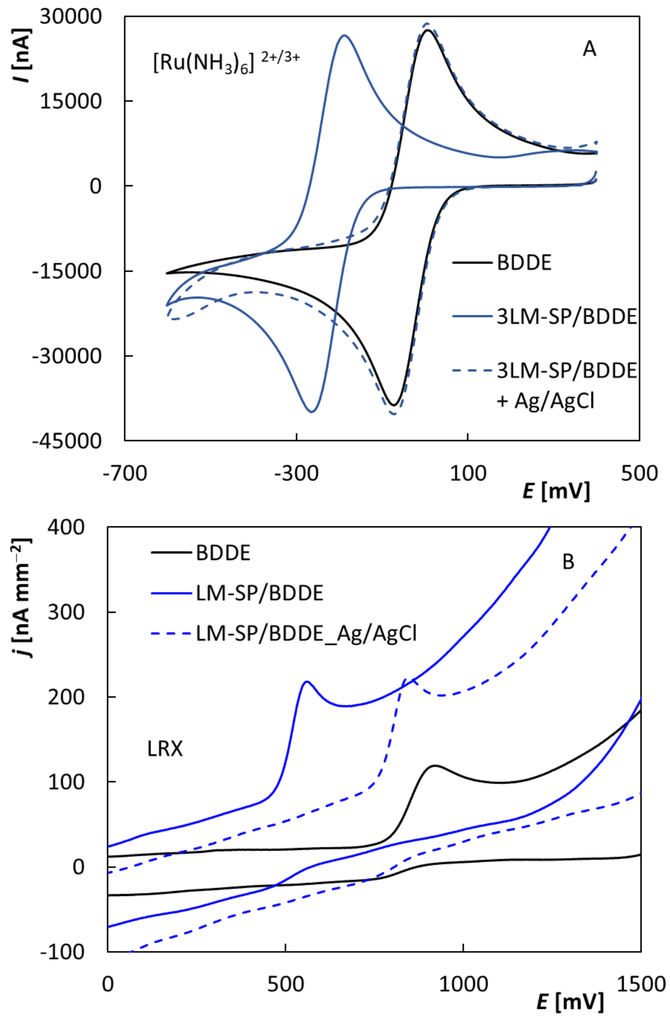

Figure S4. Cyclic voltammograms of $[Ru(NH_3)_6]^{2+/3+}$ recorded on BDDE with Ag/AgCl(KCl. sat.), on 3LM-SP/BDDE, and on 3LM-SP/BDDE with external reference electrode (Ag/AgCl(KCl sat.)) (A), and cyclic voltammograms of LRX recorded using the same 3 electrochemical cell arrangements (B) (A: electrolyte – 0.1 mol L^{-1} KCl, v = 100 mV s^{-1}, $c(Ru(NH_3)_6]^{2+/3+})$ = 2.5×10^{-3} mol L^{-1}; B: elecrolyte – BRB (pH 3), v = 100 mV s^{-1}, c(LRX) = 40 μmol L^{-1}).

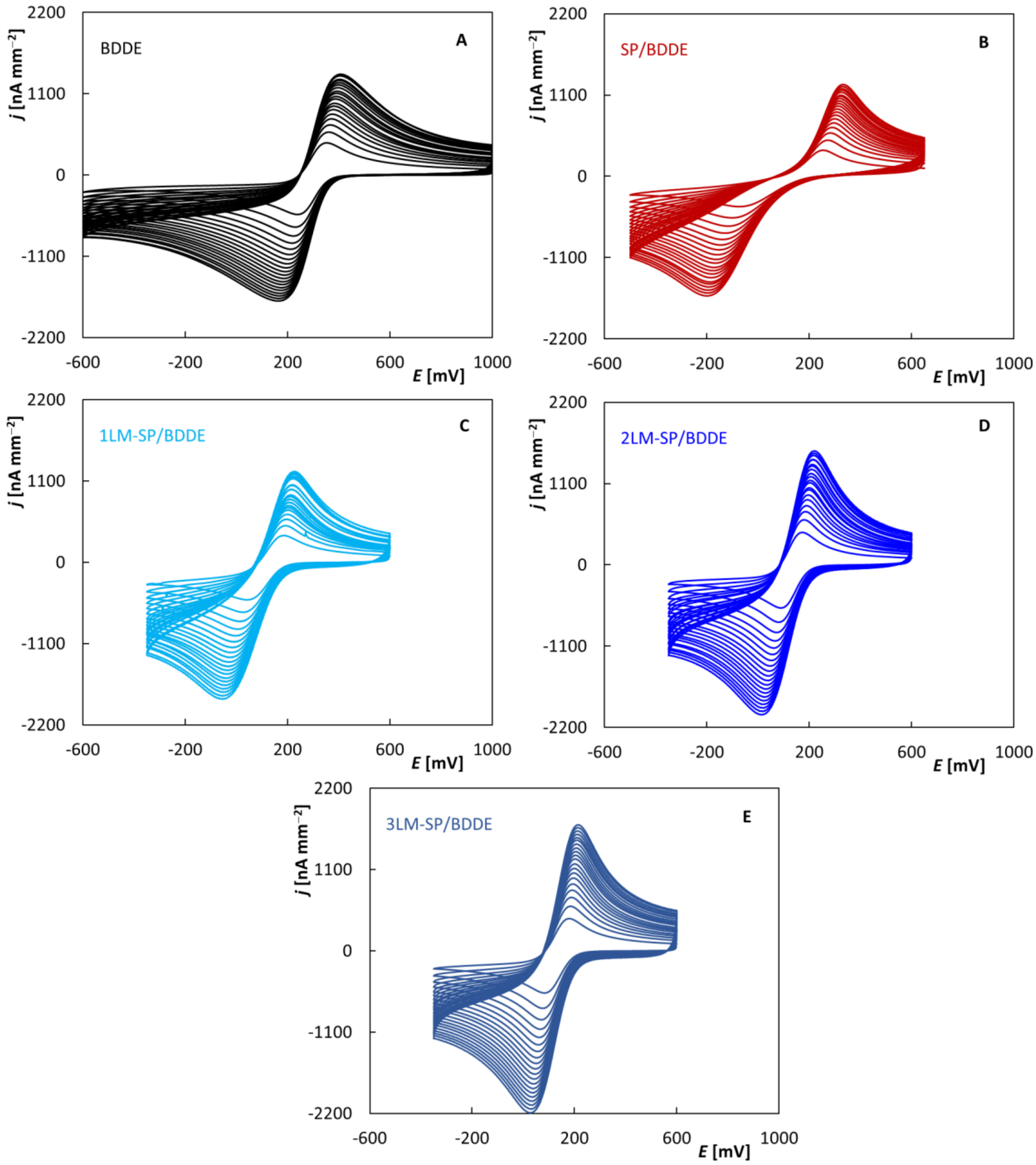

Figure S5. Cyclic voltammograms of $[Fe(CN)_6]^{4-/3-}$ recorded on tested sensors at various scan rates (electrolyte – 0.1 mol L^{-1} KCl, v = 25-500 mV s^{-1}, $c([Fe(CN)_6]^{4-/3-})$ = 6.25×10^{-4} mol L^{-1}).

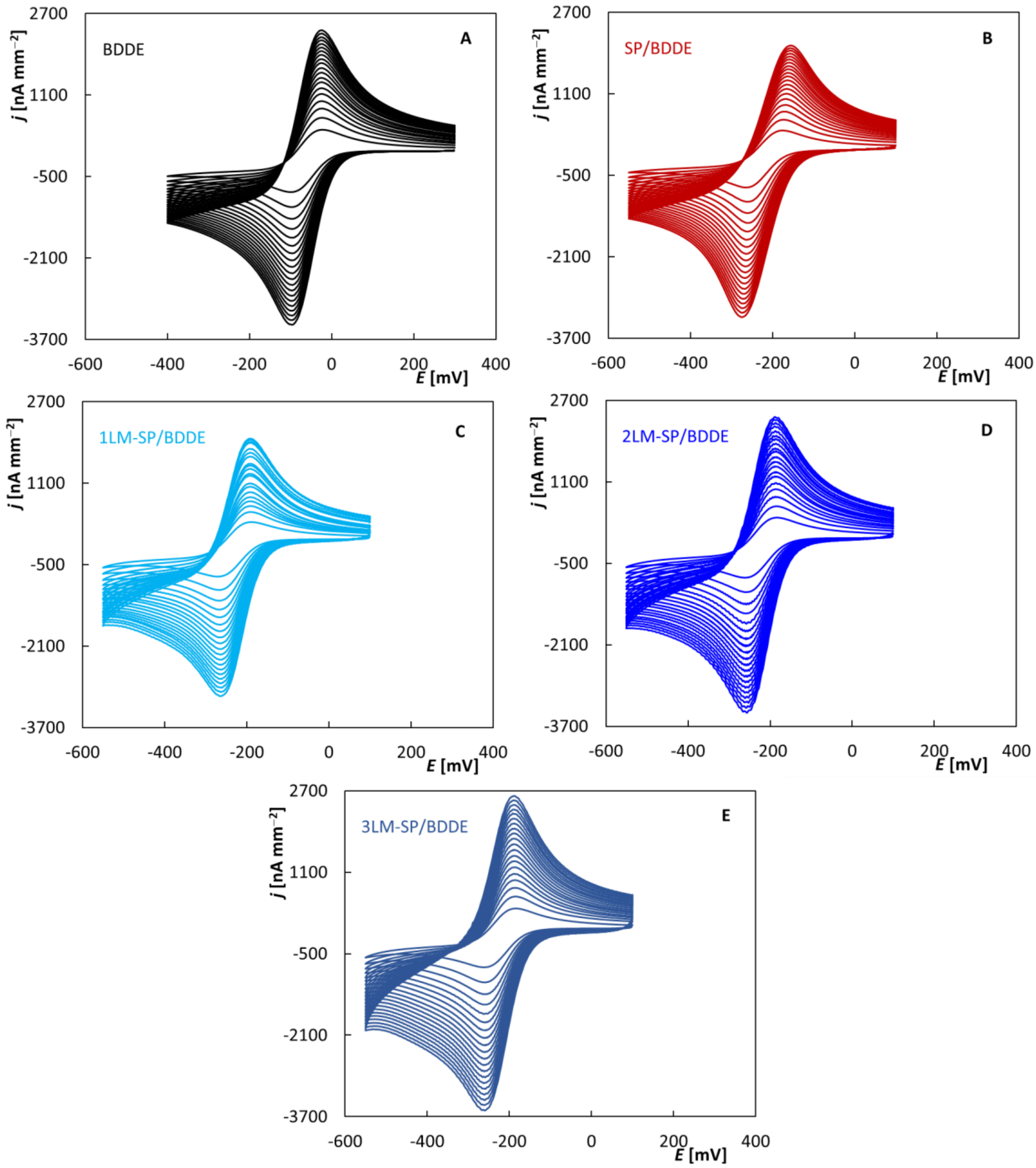

Figure S6. Cyclic voltammograms of $[Ru(NH_3)_6]^{2+/3+}$ recorded on tested sensors at various scan rates (electrolyte – 0.1 mol L^{-1} KCl, v = 25-500 mV s^{-1}, $c([Ru(NH_3)_6]^{2+/3+})$ = 6.25×10^{-4} mol L^{-1}).

Table S1. Statistical parameters of dependences of log(j_P) on log(v) for $[Fe(CN)_6]^{4-/3-}$ and $[Ru(NH_3)_6]^{2+/3+}$ (electrolyte – 0.1 mol L^{-1} KCl, v = 25-500 mV s^{-1}, $c([Fe(CN)_6]^{4-/3-})$ = 6.25×10^{-4} mol L^{-1}, $c([Ru(NH_3)_6]^{2+/3+})$ = 6.25×10^{-4} mol L^{-1}).

	Electrode	j_P	Slope [nA s mV^{-1} mm^{-2}]	Intercept [nA mm^{-2}]	r
$Fe(CN)_6]^{4-/3-}$	BDDE	a	(0.363±0.003)	(2.120±0.008)	0.9992
		c	(0.366±0.002)	(2.182±0.005)	0.9997
	SP/BDDE	a	(0.377±0.003)	(2.042±0.007)	0.9995
		c	(0.388±0.023)	(1.950±0.005)	0.9997
	1LM-SP/BDDE	a	(0.388±0.007)	(2.107±0.016)	0.9973

		c	(0.374±0.004)	(2.070±0.010)	0.9989
	2LM-SP/BDDE	a	(0.383±0.003)	(2.200±0.007)	0.9995
		c	(0.362±0.001)	(2.200±0.003)	0.9999
	3LM-SP/BDDE	a	(0.413±0.002)	(2.144±0.005)	0.9997
		c	(0.405±0.002)	(2.133±0.004)	0.9998
$Ru(NH_3)_6]^{2+/3+}$	BDDE	a	(0.489±0.001)	(2.129±0.003)	0.9999
		c	(0.509±0.001)	(2.084±0.002)	0.9999
	SP/BDDE	a	(0.469±0.001)	(2.092±0.002)	0.9999
		c	(0.521±0.001)	(2.027±0.001)	0.9999
	1LM-SP/BDDE	a	(0.489±0.004)	(2.050±0.009)	0.9994
		c	(0.510±0.004)	(2.000±0.008)	0.9996
	2LM-SP/BDDE	a	(0.507±0.006)	(2.057±0.013)	0.9989
		c	(0.527±0.002)	(2.015±0.005)	0.9998
	3LM-SP/BDDE	a	(0.525±0.006)	(2.016±0.014)	0.9989
		c	(0.533±0.001)	(2.002±0.002)	0.9999

a – anodic, c – cathodic.

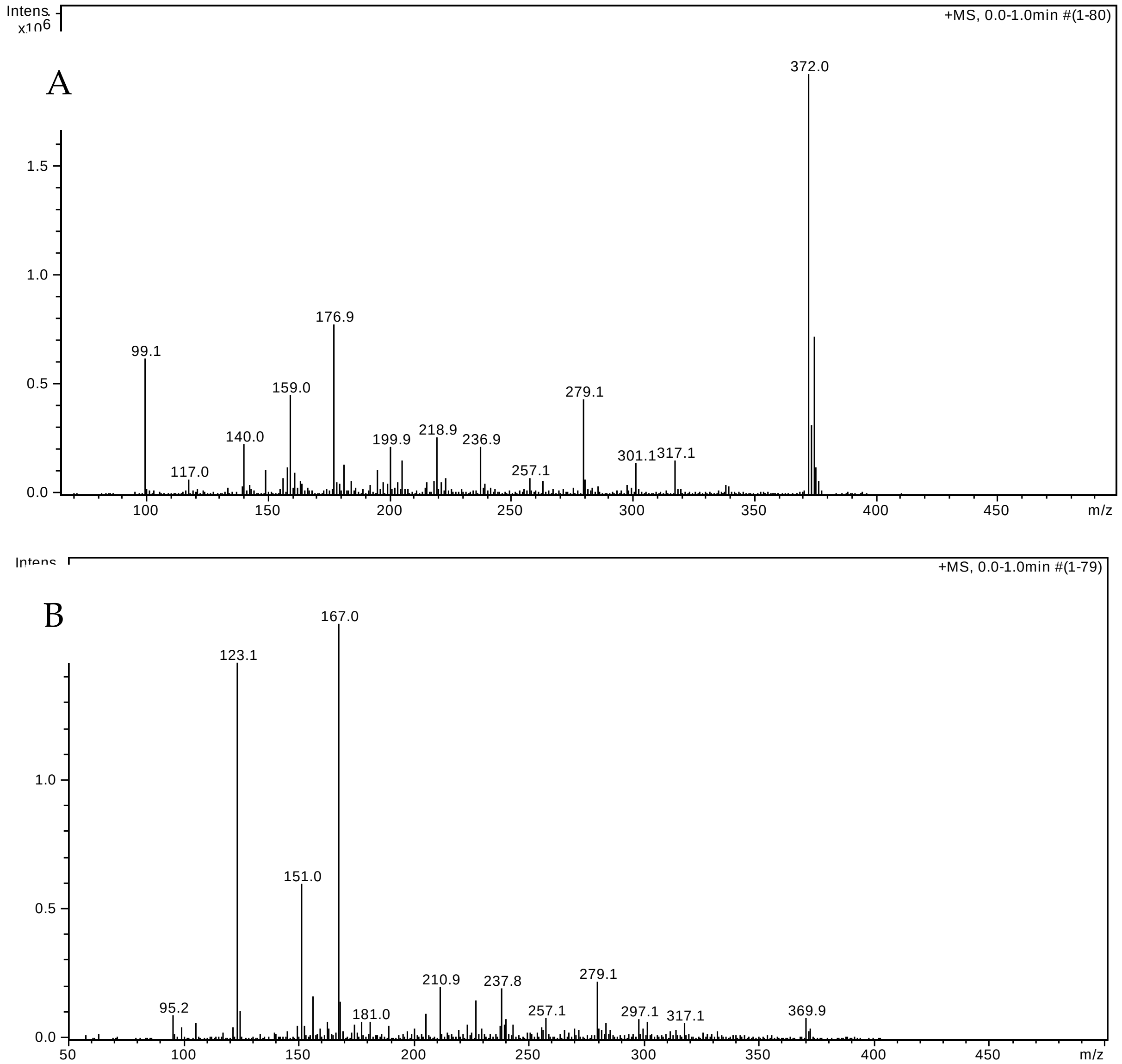

Figure S7. Mass spectra recorded in EC-MS experiment with LRX (c = 50 μmol L^{-1}) in aqueous 0.1 mol L^{-1} CH_3COOH with acetonitrile (1:1, v/v) at potential 0 mV (A) and 200 mV (B) on porous

graphite carbon electrode *vs.* Pd/H_2 reference electrode. ESI-MS conditions: nitrogen pressure in the nebulizer 15 psi, dry gas 5 L h^{-1}, dry temperature 300 °C, capillary voltage −3.5 kV, end plate offset +0.5 kV (positive mode). Ion at *m*/*z* 369.9 corresponds to $[M+H]^+$ of intermediate **II** (or the rearranged structure **VI**) in Scheme 4, ion *m*/*z* 167 belongs to $[M+H]^+$ of intermediate **IX** (ions at *m*/*z* 151, 123 and 95 are fragment ions of the ion *m*/*z* 167).

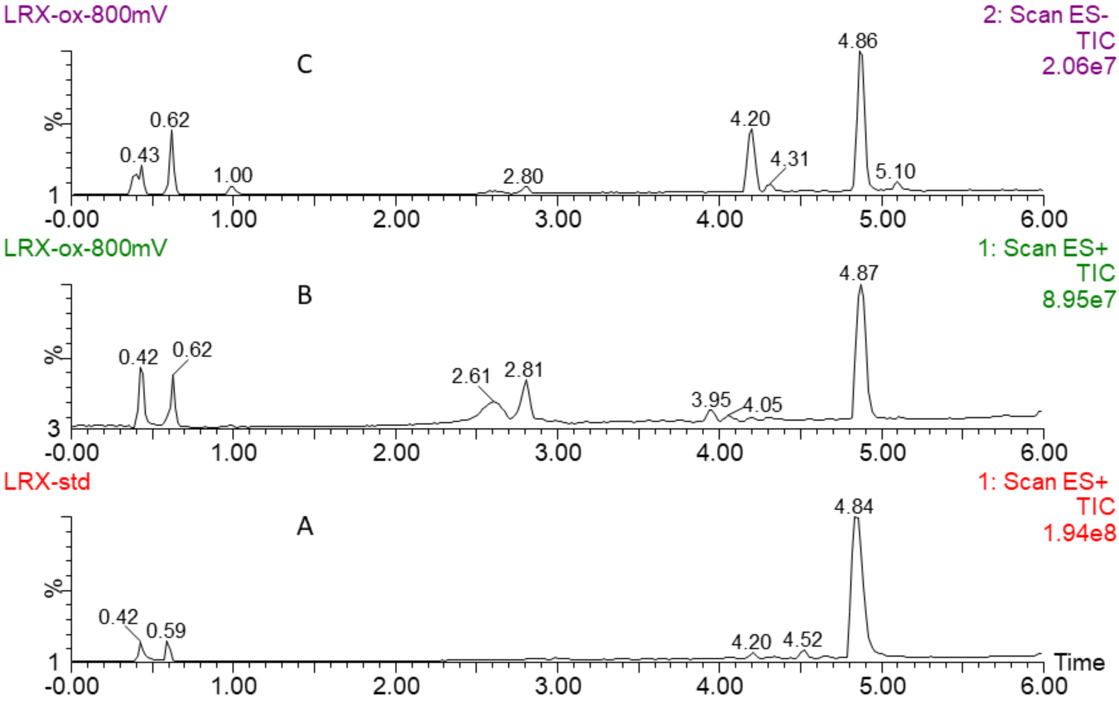

Figure S8. Total ion chromatograms of LRX solution (*c* = 50 μmol L^{-1}) in aqueous 0.1 mol L^{-1} CH_3COOH with acetonitrile (1:1, v/v) before (A) and after (B, C) electrolysis on the carbon fiber brush electrode at constant potential 800 mV vs. SCE for 60 min. Chromatograms were recorded in positive (A, B) and negative (C) ionization mode of ESI.

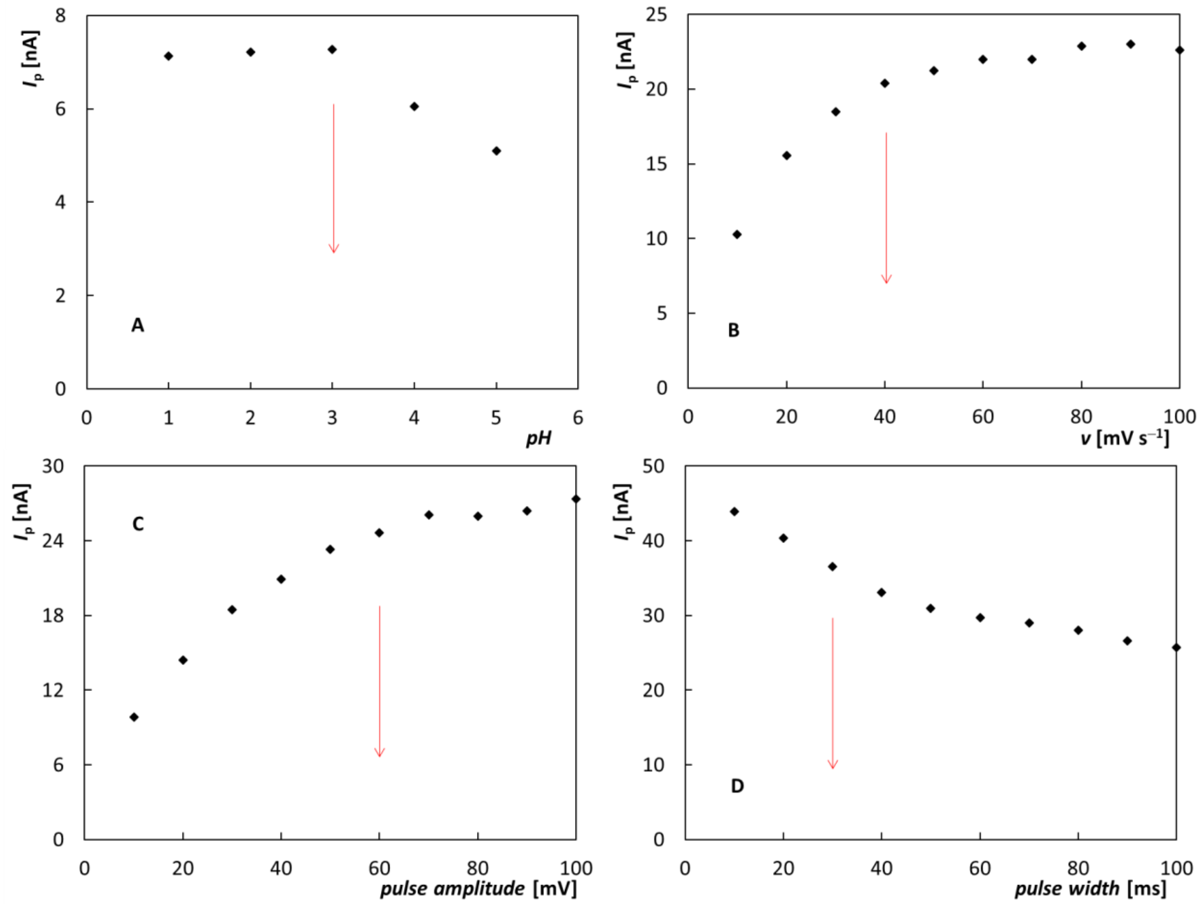

Figure S9. Optimization of DPV parameters for LRX determination using BDDE dependences of I_p on pH of supporting electrolyte (A), I_p on v (B), I_p on pulse amplitude (C), and I_p on pulse width (D) (DPV, electrolyte – BRB (pH 1-5 (A) and 3 (B-C)), E_{in} = 0 mV, E_{fin} = +2000 mV, v = 20 (A), 10-100 (B), and 40 (C, D) mV s^{-1}, pulse amplitude = +50 (A, B), 0-+100 (C), and +60 (D) mV, pulse width = 50 (A-C), and 10-100 (D) ms, c(LRX) = 2.0 μmol L^{-1}.
